# Treatment of cartilage defects by subchondral drilling combined with covering with atelocollagen membrane induces osteogenesis in a rat model

**DOI:** 10.1007/s00776-013-0379-0

**Published:** 2013-04-06

**Authors:** Michio Hamanishi, Tomoyuki Nakasa, Naosuke Kamei, Hiromi Kazusa, Goki Kamei, Mitsuo Ochi

**Affiliations:** 1 Department of Orthopaedic Surgery, Graduate School of Biomedical Sciences, Hiroshima University, 1-2-3 Kasumi, Minami-ku, Hiroshima, 734-8551 Japan; 2 Division of Regeneration and Medicine, Hiroshima University Hospital, Hiroshima, Japan

## Abstract

**Background:**

The coverage of the atelocollagen membrane at the chondral defect after subchondral drilling might improve the beneficial effects for cartilage repair because of the prevention of scattering and accumulation of cells and growth factors from bone marrow within the chondral defect. On the other hand, it might block cells and factors derived from the synovium or cause high pressure in the chondral defect, resulting in prevention of cells and growth factors gushing out from the bone marrow, which leads to disadvantages for cartilage repair.

**Method:**

We tested this hypothesis in a 2-mm-diameter chondral defect created in the articular cartilage of the patellar groove in a rat models. Defects were left untreated, or were drilled or drilled and covered with an atelocollagen membrane; healing was evaluated by histology and gene expression analysis using real-time polymerase chain reaction and immunohistochemistry.

**Results:**

Membrane coverage induced bone tissue ingrowth into the punched chondral defect. At 1 week, expression of TGFβ, Sox9, Runx2, osteocalcin, Col1a1, and Col2a1 in the drilling group was significantly higher than in the covering group. At 4 weeks, expressions of TGFβ, Runx2, and Col1a1 were all significantly higher in the drilling group, while Sox9, osteocalcin, and Col2a1 were significantly higher in the covering group. Immunohistochemistry demonstrated Sox9, osteocalcin, and type II collagen on the bony reparative tissue in the covering group.

**Conclusions:**

These results suggest that the atelocollagen membrane coverage resulted in inhibition of cartilage repair.

## Introduction

Articular cartilage has very limited healing potential, because it lacks a blood supply and is isolated from systemic regulation [1]. The most widely practiced methods of repairing defects are bone marrow stimulation techniques such as subchondral drilling, abrasion, and microfracture, procedures that aim to recruit bone marrow elements to repair cartilage defects [2–7]. Such procedures are thought to promote chondrogenesis, inducing formation of fibrous tissue, fibrocartilage, and/or hyaline cartilage by inducing migration of bone marrow mesenchymal stromal cells (MSCs) from the subchondral bone by bleeding. However, in experimental studies these techniques have resulted in the formation of fibrocartilaginous tissues [3, 7, 8]. There are several possible reasons why bone marrow-stimulating procedures do not always induce satisfactory results. One explanation is that the number of bone marrow MSCs may not be adequate to repair the lesion. Nishimori et al. [9] demonstrated that the addition of cultured bone marrow MSCs to a defect in combination with a bone marrow-stimulating procedure accelerated regeneration of articular cartilage in the defects better than the bone marrow-stimulating procedure alone.

One suggested reason why the number of bone marrow MSCs may be inadequate to repair the lesion could be that they diffuse into the joint fluid and do not remain in the chondral defect. Therefore, we estimated that the use of an atelocollagen membrane to cover a chondral defect after subchondral drilling exerts beneficial effects on cartilage repair by preventing scattering of cells and growth factors from the bone marrow and causing them to accumulate within the chondral defect. The purpose of this study was to examine the effect and the mechanism of atelocollagen membrane coverage combined with subchondral drilling on cartilage regeneration.

## Materials and methods

### Animal preparation

All procedures were performed in accordance with the Guide for Animal Experimentation, Hiroshima University, and the Committee of Research Facilities for Laboratory Animal Sciences, Graduate School of Biomedical Sciences, Hiroshima University (no. A10-97).

Male Sprague–Dawley rats (12 weeks old) were used in this study. A total of 50 knees of 26 rats were used. Before surgery, the animals were anesthetized with an intraperitoneal injection of 1 ml/kg pentobarbital sodium. The patella was everted through a medial approach. A chondral defect of 2 mm diameter was created in the articular cartilage of the patellar groove of the distal femur using a biopsy punch. The control group represented the natural course of healing of the chondral defect. In the drilling group, five holes were drilled into the cartilage using a 0.2-mm-diameter drill. In the covering group, we applied instant glue approved for clinical application (Aron Alpha A “Sankyo,” Daiichi Sankyo, Tokyo, Japan), containing α-cyanoacrylate monomer, around the drilled defect and covered it with a recombinant peptide membrane (Fujifilm, Tokyo, Japan), which has low ecotoxicology and high uniformity, created using a yeast culture technique. The membrane was a rectangle of 4 × 5 mm and 5 μm thickness. No bleeding was observed in the untreated chondral defects, while bleeding derived from the bone marrow could be observed in chondral defects treated by drilling.

In the drilling and covering group, blood clots or reparative tissue in the defect were extracted and analyzed for expression of several factors as markers of chondrogenesis and osteogenesis, and extracellular matrix was analyzed by real-time polymerase chain reaction (PCR) at 1 and 4 weeks after creation of the defects. At 4 weeks after creation of the defects, the rats were euthanized by intraperitoneal injection of a lethal dose of pentobarbital sodium. The distal femora were resected en bloc and fixed in 4 % paraformaldehyde for 24 h at 4 °C. They were then decalcified in 0.5 M EDTA, then embedded in paraffin and cut into 5-μm sections serially along the sagittal plane that included the center of the defect, and histological evaluation was performed.

### Quantitative reverse transcription polymerase chain reaction (real-time PCR)

To examine the expression of chondrogenic and osteogenic marker genes such as Col2a1, Sox9, TGFβ, Col1a1, Runx2, and osteocalcin, real-time PCR was performed using SYBR Green (Invitrogen, Carlsbad, CA, USA). Total RNA was isolated from blood clots or reparative tissue that had been homogenized on ice with Trizol reagent (Invitrogen). One μg of total RNA was reverse-transcribed using the QuantiTect^®^ Reverse Transcription Kit (Qiagen, Chatsworth, CA, USA) according to the manufacturer’s protocol. Real-time PCR was performed using a Real-time PCR System (Applied Biosystems, Carlsbad, CA) in a 20-μl PCR mixture containing 1.0-μl template cDNA, 10 μl SYBR Green mix, 1.5 μM primer, and water to adjust the final volume to 20 μl. Primer sequences are listed in Table 1.

**Table 1:** Primer sequences used for real time polymerase chain reaction (PCR)

Target genes	Primer sequence forward	Primer sequence reverse
TGF-β	5′-TATAGCAACAATTCGTGGCG-3′	5′-CAGAAGTTGGCATGGTAGCC-3′
Sox9	5-CGTCAACGGCTCCAGCA-3′	5′-TGCGCCCACACCATGA-3′
Runx2	5′-CACCCTCAAGAGCCTGAGTC-3′	5′-CAGACGGCTGAGTAGGGAAC-3′
Osteocalcin	5-GCATTCTGCCTCTCTGACCT-3′	5′-CTAAACGGTGGTGCCATAGA-3′
Col1a1	5′-TGCCGTGACCTCAAGATGT-3′	5-TGGGGI 1 IGGGCTGATGTA-3′
Col2a1	5′-CCCAGAACATCACCTACCAC-3′	5′-GGTACTCGATGATGGTCTTG-3′

All reactions were performed in triplicate in a 96-well plate and incubated at 95 °C for 10 min, followed by 40 cycles of 95 °C for 15 s and 60 °C for 1 min. The GAPDH gene was used as a control to normalize differences in total RNA levels between samples. A threshold cycle (C_T_) was observed in the exponential phase of amplification, and quantification of relative expression levels was performed using standard curves for target genes and the endogenous control. Geometric means were used to calculate the ΔΔC_T_ (delta–delta C_T_) values and expressed as 2^−ΔΔCT^. The value of each control sample was set at 1 and used to calculate the fold change of target genes.

### Histological evaluation

For histological evaluation of cartilage regeneration, sections were stained with safranin-O/fast green. The specimens were graded semi-quantitatively by two observers who were not aware of the source of cartilage. The grading scale was based on filling of the defect (0–4 points), any reconstitution of the osteochondral junction (0–2 points), matrix staining (0–4 points), and cell morphology (0–4 points) as described by Pineda et al. [10] (Table 2).

**Table 2:** Histologic grading of the osteochondral defects (Pineda et al. [11])

Category	Points
Cell morphology	
Hyaline cartilage	0
Mostly hyaline cartilage	1
Mostly fibrocartilage	2
Mostly non-cartilage	3
Non-cartilage only	4
Matrix staining (metachromasia)	
Normal (compared with host adjacent cartilage)	0
Slightly reduced	1
Markedly reduced	2
No metachromatic stain	3
Surface regularity	
Smooth (>3/4)	0
Moderate (>1/2–3/4)	1
Irregular (1/4–1/*2)*	2
Severely irregular (<1/4)	3
Thickness of cartilage	
>2/3	0
1/3–2/3	1
<1/3	2
Integration of donor with host adjacent cartilage	
Both edge integrated	0
One edge integrated	1
Neither edge integrated	2
Total maximum	14

For immunohistological evaluation, sections were mounted onto poly-l-lysine-coated glass slides and immersed in 0.3 % H_2_O_2_ to block endogenous peroxidase activity. The sections were blocked with normal goat serum and then incubated with mouse monoclonal antibodies directed against type II collagen and Runx2, and rabbit monoclonal antibodies directed against TGF-β, Sox9, osteocalcin, and type I collagen (Fuji Chemipha, Toyama, Japan). The reaction for visualization was performed using an avidin-biotin peroxidase system (Vectastain Elite ABC kit; Vector Laboratories, Burlingame, CA, USA), and color was developed with a freshly prepared diaminobenzidine solution.

### Statistical analysis

One-way analysis of variance (ANOVA) followed by Tukey’s post hoc analysis was used to compare Pineda’s scales among the three groups. The Mann-Whitney *U* test was used to compare gene expression between two groups. *P* values less than 0.05 were considered to be statistically significant. All statistical analyses were performed on a personal computer using the Stat View statistical package version 5.0(Abacus Concepts, Berkeley, CA, USA).

## Results

### Gross appearance

**Fig. 1:**
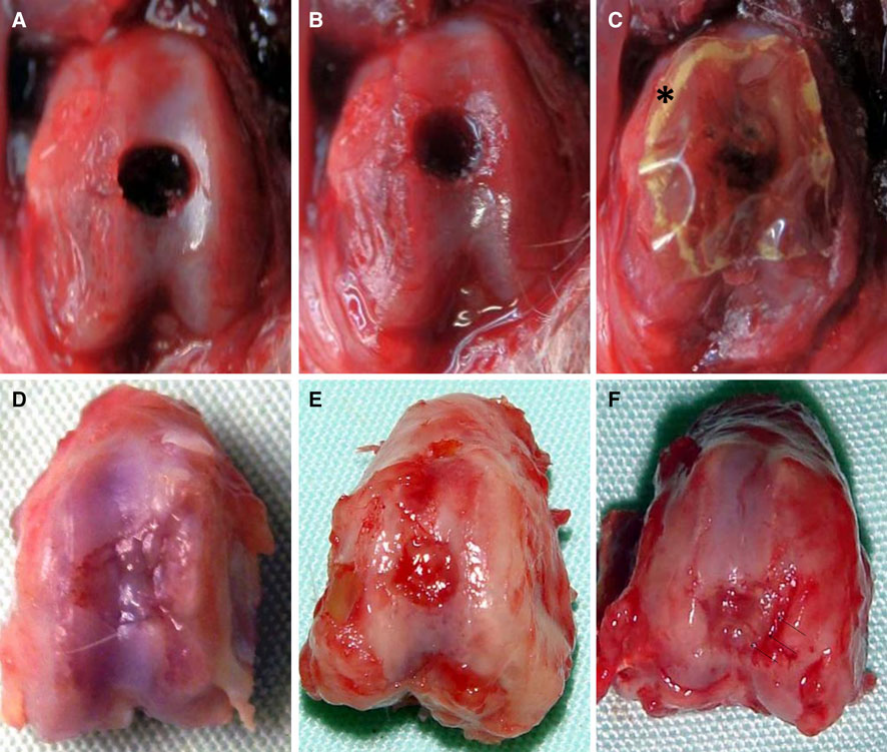
Representative pictures of chondral defects at 1 week and 4 weeks after the creation of models. **a** Control group (1 week) represents the natural course of healing of the chondral defect. **b** Drilling group (1 week); five holes were drilled into the chondral defect using a 0.2-mm-diameter drill. **c** Covering group (1 week); instant glue suitable for clinical use was applied around the drilled defect, and a recombinant peptide membrane was used to cover it. **d** Control group (4 weeks); ingrowth of fibrous tissue was observed. No cartilage was observed growing into the margins of the defect. **e** Drilling group (4 weeks); ingrowth of hyaline cartilage could be observed in a deep layer of fibrous tissue. **f** Covering group; ingrowth of bony tissue was observed, while no growth of hyaline cartilage into the margins of the defect could be seen

At 1 week, blood clots were present in the chondral defects of both the drilling and the covering groups, while the cartilage defect in the control group appeared to be empty. In the covering group, the atelocollagen membrane covering the chondral defect still remained (Fig. 1a–c). At 4 weeks, all models showed ingrowth of reparative tissue into the defects (Fig. 1d–f). In the control group, the defect margins were clearly recognizable. The defect was filled with fibrous tissue, and its surface was rough and depressed (Fig. 1d). In the drilling group, the margins of the cartilage defects were also clearly recognizable, and the defect surface was depressed; however, reparative tissue filling the defect was smooth, with an appearance like the normal surface of joint cartilage (Fig. 1e). In the covering group, the defect margins were uneven, and bony spurs were observed in the defect. The surface of the cartilage around the defect showed osteoarthritic changes with degeneration of cartilage and spur formation (Fig. 1f).

### Histological evaluation

**Fig. 2:**
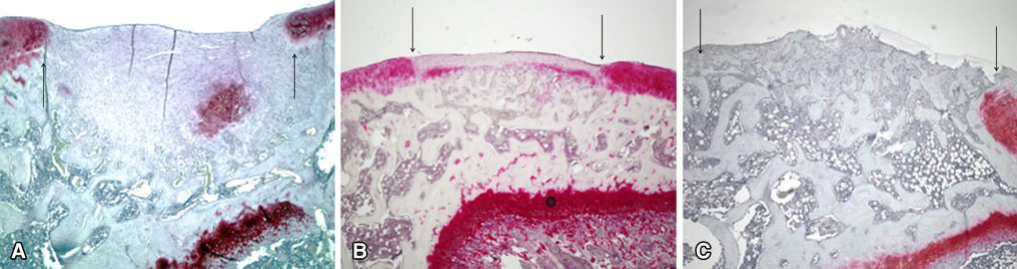
Safranin O/fast green staining of chondral defect sections at 4 weeks after the creation of models. **a** Control group; ingrowth of fibrous tissue could be seen. No cartilage was observed growing into the margins of the defect. **b** Drilling group; ingrowth of hyaline cartilage could be observed in the deep layer of fibrous tissue. **c** Covering group; there was ingrowth of bony tissue into the defect, but no growth of hyaline cartilage could be observed. Focus; ×40

In the control group, no staining with safranin O was observed in the chondral defect area; instead fibrous tissue filled the chondral defect (Fig. 2a). The histological score of all the control samples was 11 points. In the drilling group, the margins of the defect were clearly recognizable and ingrowth of hyaline cartilage could be observed in the deep layer of fibrous tissue in the defect (Fig. 2b). The mean histological score was 6.2 ± 0.6 points. In the covering group, the margins of the defect were irregular because of bone formation. Virtually no hyaline cartilage was observed in the margins of the defect, while in the chondral defect area, abundant bone tissue was observed (Fig. 2c). The mean histological score was 12.7 ± 0.6 points. The histological score in the drilling group was therefore significantly better than in the other two groups. In contrast, the histological score in the covering group was significantly worse than that in the other two groups (Table 3).

**Table 3:** Summary of gross appearance, PCR analysis, histological, and immunohistochemical results

Control (*n* = 5)		Drilling (*n* = 5)		Covering (*n* = 5)	
Gross appearance at 4 weeks	Rough and depressed	Smooth		Uneven, bony spur	
Pineda’s score	11 ± 0*	6.2 ± 0.6*		12.7 ± 0.6*	
Real time PCR		1 week	4 weeks	1 week	4 weeks
TGFβ		1.9 ± 0.4*	1.2 ± 0.4*	1.2 ± 0.6*	0.4 ± 0.3*
Sox9		9.7 ± 9.3*	0.7 ± 0.3*	1.6 ± 0.9*	0.4 ± 0.2*
RUNX2		1.5 + 0.9*	1.1 ± 1.3*	0.6 ± 0.3*	0.7 ± 0.4*
Osteocalcin		6.1 ± 6.0*	1.1 ± 0.3*	0.5 ± 0.4*	2.3 ± 0.4*
Collal		2.6 ± 1.6*	0.9 ± 0.2*	1.9 ± 0.6*	0.3 ± 0.2*
Col2al		2.9 ± 3.3*	1.0 ± 0.2*	1.4 + 1.7*	2.2 ± 0.5*
Immunohistological results					
The distribution of positive immunoreactivity					
TGFβ	No immunoreactivity	Deep layer of fibrous reparative tissue above the area of ingrowth of hyaline cartilage		No immunoreactivity	
Sox9	Deep layer of reparative tissue	Area of hyaline cartilage ingrowth		Bony reparative tissue	
Runx2	No immunoreactivity	Area of hyaline cartilage ingrowth		Shallow layer of reparative tissue	
Osteocalcin	Shallow layer of reparative tissue	Area of hyaline cartilage ingrowth		All area of reparative tissue	
Collal	No immunoreactivity	Fibrous reparative tissue		Shallow fibrous reparative tissue	
Col2al	No immunoreactivity	Area of hyaline cartilage ingrowth		Bony reparative tissue	

### Gene expression analysis

**Fig. 3:**
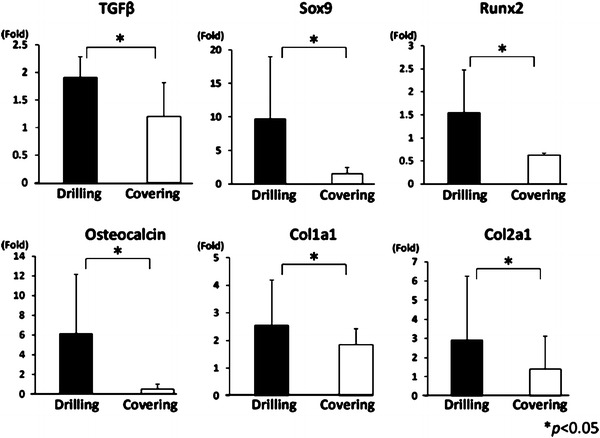
Real-time PCR analysis at 1 week after creation of the defects. The expressions of TGFβ, SOX9, RUNX2, osteocalcin, Col1a1, and Col2a1 were significantly higher in the drilling group (*P* < 0.05)

**Fig. 4:**
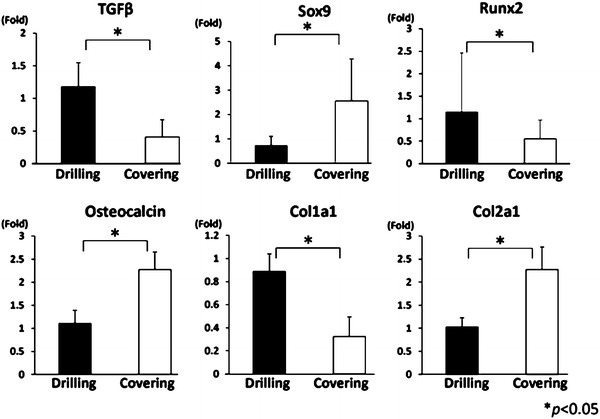
Real-time PCR at 4 weeks after creation of the defects. The expressions of TGFβ, SOX9, RUNX2, and Col1a1 were significantly higher in the drilling group. Conversely, the expressions of osteocalcin and Col2a1 were significantly higher in the covering group (*P* < 0.05)

At 1 week, real-time PCR analysis revealed that expressions of TGFβ, Sox9, Runx2, osteocalcin, Col1a1, and Col2a1 in the drilling group were all significantly higher than in the covering group (Fig. 3). At 4 weeks, the expressions of TGFβ, Runx2, and Col1a1 were significantly higher in the drilling group, while the expressions of Sox9, osteocalcin, and Col2a1 were significantly higher in the covering group (Fig. 4).

### Immunohistological assessments

**Fig. 5:**
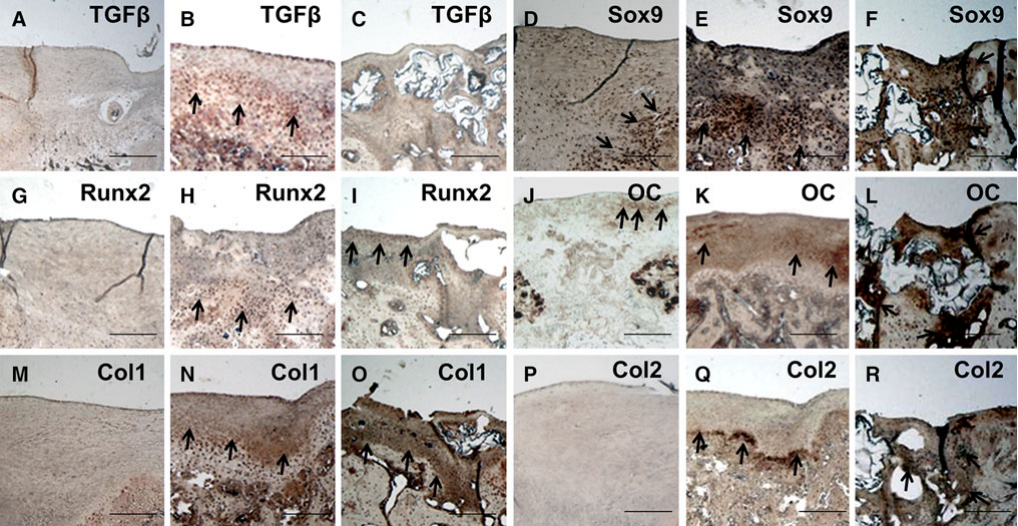
Immunohistochemistry at 4 weeks after creation of the defects. **a**, **b**, **c** TGF-β; no immunoreactivity was observed in the reparative tissue in the control group. Positive immunoreactivity was observed in the deep layer of fibrous reparative tissue above the area of ingrowth of hyaline cartilage stained with safranin O/fast green in the drilling group, and no immunoreactivity was observed in the covering group. **d**, **e**, **f** Sox9; immunopositive cells were observed in the deep layer of fibrous reparative tissue in the control group, on the area of hyaline cartilage ingrowth in the drilling group, and in bony reparative tissue in the covering group. **g**, **h**, **i** Runx2; no immunopositive cells in the reparative tissue were observed in the control group. Immunopositive cells were observed in the area of hyaline cartilage ingrowth in the drilling group and in the shallow layer of reparative tissue in the covering group. **j**, **k**, **l** Osteocalcin; positive immunoreactivities were observed in the shallow layer of reparative tissue in the control group, in all areas of reparative tissue in both the drilling and the covering groups. Especially strong staining was observed in the bony reparative tissue in the covering group. **m**, **n**, **o** Type I collagen; no immunoreactivity was observed in the reparative tissue in the control group. Positive immunoreactivity was observed in the fibrous reparative tissue in the drilling group and in the shallow fibrous reparative tissue in the covering group. **p**, **q**, **r** Type II collagen; no immunoreactivity was observed in the reparative tissue in the control group. Positive immunoreactivity was observed in the area of hyaline cartilage ingrowth in the drilling group and in bony reparative tissue in the covering group. Focus, ×40. *Scale bar*; 200 μm

Immunohistochemistry at 4 weeks after creation of the defects showed positive immunoreactivity of TGFβ in the deep layer of fibrous reparative tissue above the area of ingrowth of hyaline cartilage stained with safranin O/fast green in the drilling group (Figs. 2b, 5b), but no positive immunoreactivity in the covering group (Fig. 5c). However, Sox9-immunopositive cells were observed in the area of hyaline cartilage ingrowth in the safranin O/fast green-stained area in the drilling group (Fig. 2b, 5e) and the bony reparative tissue in the covering group (Fig. 5f). Runx2-immunopositive cells were observed in the safranin O/fast green-stained area of hyaline cartilage ingrowth in the drilling group (Figs. 2b, 5h) and in the shallow layer of reparative tissue in the covering group (Fig. 5i). Positive staining for osteocalcin was observed in all areas of reparative tissue in the covering group. In the drilling group, the area of hyaline cartilage showed immunoreactivity for osteocalcin (Figs. 2b, 5k). Particularly strong osteocalcin staining was observed in bony reparative tissue in the covering group (Fig. 5l). Positive immunoreactivity for type I collagen was observed on the fibrous reparative tissue in the drilling group (Fig. 5n) and in the shallow fibrous reparative tissue in the covering group (Fig. 5o), while positive immunoreactivity for type II collagen was observed in the area of safranin O/fast green-stained hyaline cartilage ingrowth in the drilling group (Figs. 2b, 5q) and in the bony reparative tissue in the covering group (Fig. 5r).

These results are summarized in Table 3.

## Discussion

We had expected that atelocollagen membrane coverage of the drilled chondral defect might exert beneficial effects on cartilage repair because of the accumulation of cells and growth factors derived from bone marrow in the chondral defect. However, in contrast to our expectations, the findings of this study demonstrate that covering the chondral defect induced the ingrowth of bone-like tissue instead of cartilage regeneration in the chondral defect area.

At 1 week after creation of the defects, the mRNA expression levels of TGF-β, Sox9, Runx2, osteocalcin, Col2a1, and Col1a1 in the covering group were significantly lower than in the drilling group. The expression pattern of these factors might contribute to poor chondral repair caused by coverage of the chondral defect. On the other hand, the mRNA expression levels of osteocalcin were significantly higher in the covering group at 4 weeks after creation of the defect. Osteocalcin promotes the differentiation and function of osteoblasts and maturation of bone cells [11]. Therefore, the high mRNA expression of osteocalcin and its positive immunoreactivity on the bony reparative tissue in the covering group suggest that coverage of the cartilage defect induced the growth of bony tissue into the chondral defect. Expressions of both TGF-β and Runx2 were suppressed in the chondral defect covered with atelocollagen membrane both at 1 week and 4 weeks. TGF-β inhibits differentiation of osteoblasts by inhibiting the function of Runx2 [12–14] and promotes the proliferation of chondrocytes through the MEK-ERK-Elk1 signaling pathway [15, 16]. Runx2 promotes the differentiation of immature chondrocytes into mature chondrocytes and meanwhile inhibits differentiation of immature osteoblasts into mature osteoblasts [17, 18]. Therefore, low levels of TGF-β and Runx2 might be disadvantageous for chondrogenesis in the cartilage repair process.

The technique of subchondral drilling promotes chondrogenesis with the formation of fibrous tissue, fibrocartilage, and/or hyaline cartilage by inducing the influx of bone marrow mesenchymal stromal cells from the subchondral bone by bleeding. However, in previous experimental studies this method has been shown to result in the formation of fibrocartilage with no formation of hyaline cartilage [3, 6, 7]. Mienaltowski et al. [19] created a chondral defect and performed microfracture of the subchondral bone in the medial femoral condyles of Quarter horses and compared the expression of biomarkers in the reparative tissue with that in normal cartilage using real-time PCR. They found that the expression of Col1a1 was higher in the reparative tissue, while the expressions of Col2a1 and Sox9 were higher in normal cartilage. These results of reparative tissue vs. normal cartilage are similar to our results comparing the drilling group and the covering group, leading us to conclude that the pattern of expression of biomarkers is more natural in the covering group.

There are several concerns associated with this study. The first relates to the effect of the glue used in this study on normal cartilage. The glue we used (Aron Alpha A) is frequently used in the clinical setting. Several authors have demonstrated fixation of osteochondral fragments using Aron Alpha A and reported that there were no foreign matter reactions in the cartilage [20, 21]. We tried gluing the recombinant peptide membrane to normal cartilage of the distal femur using Aron Alpha A as a preliminary experiment and concluded that it could be used safely for this study. Second, there was the possibility of foreign matter reactions caused by the membrane. Recombinant peptide membranes are created using a yeast culture technique and have low ecotoxicity. In the preliminary experiment described above, there were no foreign matter reactions, such as synovitis, and this suggested that recombinant peptide membranes can be used safely in the joint. Third, rat chondrocytes and human chondrocytes are very different concerning structure and regenerative potential. Therefore, the results of our study cannot be applied directly to humans. Fourth, there was uncertainty concerning how long the recombinant peptide membrane was retained on the surface of the cartilage and played a role in blocking cell migration. Therefore, we investigated this by exposure of the knee in the covering group at 1, 2, 3, and 4 weeks after the operation. This showed that the membrane was retained completely at 1 and 2 weeks. However, breaks appeared in the membrane and it was only partially retained, exposing the defect, at 3 weeks. At 4 weeks, the membrane had become completely detached from the surface of the cartilage (data not shown). To further confirm the integrity of the membrane, at 1 and 2 weeks, 1 ml of indigotindisulfonate sodium was injected into the joint space, and this was then exposed 1 day after injection to confirm whether the membrane was able to block movement of the joint fluid. This investigation showed that the defect was not stained at 1 or 2 weeks (data not shown). According to these results, the recombinant peptide membrane was able to block movement of joint fluid 2 weeks after the operation in our study.

In our study, the atelocollagen membrane blocked the entry of cells and factors derived from the synovium. In addition, this procedure caused high pressure in the chondral defect and resulted in prevention of cells and growth factors gushing out from bone marrow. These mechanisms may have an inhibitory effect on cartilage repair. Christoph et al. reported a beneficial effect of coverage of a microfractured chondral defect using a cell-free polyglycolic acid scaffold [22]. Thore et al. used a cell-free resorbable polymer felt as the covering material and reported excellent clinical results [23]. In these studies, however, the covering materials acted as scaffolds for blood clot formation but did not block the influx of joint fluid because of their felt-like structure. The synovial fluid contains prolactin, and it induces type II collagen and synthesis of proteoglycans [24]. Furthermore, the synovial fluid contains TGFβ, and its concentration increases during the tissue repair process [25]. In the present study, the synovial fluid was not able to permeate the recombinant peptide-covering membrane, and these factors were therefore unable to cause a beneficial effect on chondrogenesis.

The recombinant peptide membrane exerted a blocking effect for 2 weeks after creation of the cartilage defect; consequently, synovial fluid might have influenced healing of the defect thereafter. However, the directivity of tissue regeneration is decided within 2 weeks of creation of the cartilage defect [26]. Therefore, the factors derived from synovial fluid couldn’t have a beneficial effect on cartilage repair after 2 weeks. Furthermore, the stimulation of TGFβ promotes the expression of Sox9, and Sox9 promotes the primary stage and inhibits the late stage of chondrocyte differentiation [27, 28]. Therefore, we surmised that low expression of Sox9 at 1 week, influenced by coverage, caused inhibition of the primary stage of chondrocyte differentiation, while high expression of Sox9 at 4 weeks, induced by stimulation by TGFβ contained in the synovial fluid, caused inhibition of the late stage of chondrocyte differentiation.

In order to benefit from such an effect on cartilage repair by covering the chondral defect, it might be necessary to use a membrane that allows permeation of cytokines derived from the synovium.

In conclusion, coverage of chondral defects with atelocollagen after subchondral drilling inhibited cartilage repair.
